# Efficacy, Safety and Biological Characteristics of Formulation Based on Essential Oil Against Co‐Infections of Herpes Simplex Virus‐1 and *Candida albicans*

**DOI:** 10.1002/mbo3.70199

**Published:** 2026-01-14

**Authors:** Maura Di Vito, Domiziana Coggiatti, Marilena La Sorda, Stefania Garzoli, Giulia Lombardini, Debora Talamonti, Scilla Pizzarelli, Abdesselam Zhiri, Margherita Cacaci, Riccardo Torelli, Maurizio Sanguinetti, Francesca Bugli

**Affiliations:** ^1^ Dipartimento di Scienze Biotecnologiche di Base, Cliniche Intensivologiche e Perioperatorie Università Cattolica del Sacro Cuore Rome Italy; ^2^ Dipartimento di Scienze di Laboratorio e Infettivologiche Fondazione Policlinico Universitario Agostino Gemelli IRCCS Roma Italy; ^3^ Dipartimento di Chimica e Tecnologie del Farmaco Università di Roma Sapienza Rome Italy; ^4^ Knowledge Unit (Documentation, Library) Istituto Superiore di Sanità Rome Italy; ^5^ R&D Department Pranarom international 37, Avenue des Artisans Ghislemghien Belgium; ^6^ Plant Biotechnology Research Unit Université Libre de Bruxelles (ULB) Brussels Belgium

**Keywords:** antifungal activity, anti‐inflammatory activity, antiviral activity, basophil activation test, co‐infection

## Abstract

Oral coinfections involving herpes simplex virus (HSV) and *Candida albicans* can potentially interact and exacerbate each other. Starting from bibliographical investigation, this study aimed to examine the effectiveness of some essential oils (EOs), and their commercial formulations, both against *C. albicans* and HSV‐1, identifying their antimicrobial, anti‐inflammatory and allergenic potential. A preliminary review examined essential oils' efficacy against HSV‐1. Broth microdilution tested 14 EOs, a commercial formulation (LA), and a homemade one (MIX) against three fungal strains. The quality of LA, MIX and single EOs was assessed by Solid Phase Microextraction (SPME) sampling coupled with gas chromatography‐mass spectrometry (GC‐MS) analysis. To assess the allergenic activity of MIX, LA, and single EOs a Basophil Activation Test (BAT) was standardized. ELISA tests were done to evaluate the anti‐inflammatory activity. The bibliographic search highlighted seven EOs active against HSV‐1. Four EOs showing strong antifungal activity were blended, following IFRA lip‐application limits, to create a formulation (MIX) for comparison with a commercial herpes treatment (LA). Formulations were active against HSV‐1, able to modulate the expression of pro (TNF‐α_LA_ = −29.7% and TNF‐α_MIX_ = −33.6%) and anti‐inflammatory (IL‐1β _LA_ = −50.0% and IL‐1β _MIX_ = −25.0%) cytokines and no allergenic. MIX reinforces target cells and blocks viral entry, while LA also limits intracellular replication. EO‐based formulations show promise for managing HSV‐1 and Candida co‐infections, offering antiviral, antifungal, and anti‐inflammatory effects. BAT results indicate no basophil activation at tested concentrations, supporting their safety.

## Graphical abstract

Essential oils with anti‐HSV‐1 activity were identified through literature review and tested against *Candida* to develop a formulation for co‐infections. The blend was assessed for anti‐inflammatory properties, allergenic potential via standardized BAT, and overall antiviral efficacy.

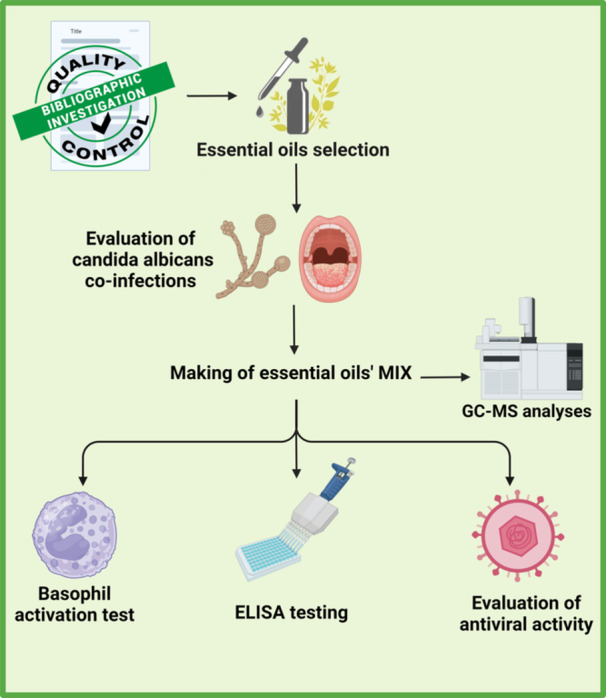

## 1 Introduction

The mouth harbors a highly diverse natural microbiota, ranking second only to the large intestine in microbial complexity (Santosh and Reddy 2017). Microbial colonization predominantly originates from the tongue, which serves as the primary reservoir for saliva‐borne microbes and as a major site for oral microbial proliferation (Fakhruddin et al. 2023). Various bacterial, viral, and fungal agents have the potential to affect the skin and mucous membranes within the orofacial region, breaching natural barriers and inducing lesions of varying severity, and some of which may be life threatening. The composition of the oral microbiota is influenced by both local and systemic factors, including genetics, diet, oral hygiene, dentures, and medications (Santosh and Reddy 2017). Orofacial infections can arise under specific conditions such as, antibiotic use, trauma, and xerostomia, leading to oral cavity damage and potential spread to adjacent tissues, culminating in systemic infections (Lalla et al. 2013). Unlike diseases of the teeth and gums, which are primarily caused by bacteria, diseases of the oral mucosa rarely have a bacterial origin (Villar and Dongari‐Bagtzoglou 2021). More than 150 species of *Candida* have been identified so far, with *Candida albicans* as the one most commonly associated with human infections (Lu 2021). Responsible for over 80% of human candidiasis cases, *C. albicans* is the most commonly detected yeast‐like fungus in the oral cavity (Martins et al. 2011). The occurrence of oral candidiasis is approximately 4% among the general population, with a higher frequency observed in people with conditions such as diabetes mellitus, immunodeficiency, or following antibiotic use (Lu 2021). These infections occur due to the proliferation and increased virulence of various *Candida* species under specific conditions of oral dysbiosis, driven by changes in the diversity and composition of the oral microbiome influenced by systemic and local factors (Contaldo 2023).

Data published by the World Health Organization indicates that approximately 4 billion of people suffer from herpetic infections. Herpes simplex virus (HSV) is a virus that belongs to the Herpesviridae family, Alpha herpesvirus subfamily and to the simplex virus genus. Specifically, the type 1 of HSV (HSV‐1) is the most diffused (3.7 billion cases) and causes orofacial, muco‐cutaneous, and encephalitis infections. Whereas the type 2 (HSV‐2, 400 billion cases) generally causes urogenital infections (Sharifi‐Rad et al. 2017; Chang et al. 2023). However, an increase in genital and neonatal herpes caused by HSV‐1 has recently been reported (Marchi et al. 2017). It is estimated that approximately 90% of people in the world are infected with at least one of these viruses (Looker et al. 2015). Although HSV is responsible for generally non‐serious infections, it is among the most widespread viruses and can cause serious manifestations in weakened individuals (Plotkin et al. 2016a). Oral coinfections involving HSV and *C. albicans* are common (Chen et al. 2011). These coinfections can occur due to various factors including compromised immune function, oral hygiene practices, and medical conditions. When these two pathogens coexist in the oral cavity, they can potentially interact and exacerbate each other's symptoms. For instance, HSV infection can compromise the integrity of the oral mucosa, making it more susceptible to *Candida* overgrowth. Conversely, *Candida* infection can cause inflammation and damage to the oral tissues, providing an opportunity for HSV to reactivate and cause recurrent lesions (Plotkin et al. 2016a; Ascione et al. 2017). Recent research has shown that HSV‐1 and coxsackie virus B5 can be effectively trapped and protected by *Candida* biofilms. Additionally, HSV‐1 has been found to enhance *C. albicans* adherence, biofilm formation, and resistance to antifungal defenses (Ascione et al. 2017). Another report illustrates the capacity of HSV to regulate the adherence of various opportunistic pathogens that coexist within the same microbiota. The study highlights that both HSV‐1 and HSV‐2 can influence the adherence of *Candida* and bacteria to HeLa229 cells, potentially through the alteration of heparan sulfate cell surface display by HSV (Plotkin et al. 2016a). Control of oral coinfections between HSV and *C. albicans* require careful management to alleviate symptoms and prevent complications, particularly in individuals with compromised immune function.

To date, there are no HSV vaccines available, and antiviral drugs may not always be effective due to resistant virus variants and biological interactions that reduce drug efficacy (Chang et al. 2023). Moreover, the adverse effects of antiviral drugs can compromise patient health (Vonberg et al. 2023). This prompts researchers to explore new molecules (e.g., natural compounds) and methods (e.g., physical treatments) with antiviral activity as alternative treatments for HSV infections. The natural world offers a significant source of potential resources targeting viral infections. Starting from a careful bibliographic investigation about the anti‐HSV‐1 activity of EOs, this study aimed to examine the effectiveness of some Mediterranean EOs, and their commercial formulations, against both *C. albicans* and HSV‐1. The study also explored their anti‐inflammatory effects, crucial for successful anti‐herpetic treatment. Concerns about the allergenic potential of essential oils (EOs) persist, with conflicting literature data and a lack of standardized tests for systemic allergenicity assessment. Currently, only prick tests are available to evaluate a limited number of EO active compounds. For the first time, this study documents the use of a basophil activation test (BAT) to assess the allergenic potential of individual EOs or mixtures for their topical application.

## 2 Materials and Methods

### 2.1 Bibliographic Search

A preliminary analysis of the literature was done to investigate the state of the art (until May 2024) relating to the efficacy of EOs of the treatment of HSV‐1.

*Eligible criteria*: Articles related to the use of EOs against HSV‐1 were included in the research. *Information source*: MEDLINE and Embase were used to perform a bibliographic analysis. *Search strategy*: The search was limited to human studies published in English, Spanish, French, or Italian, but no date limits were applied. The search strategy was developed with subject headings from controlled vocabulary that is MeSH terms (MH) and Emtree terms, combined with free‐text words in order to identify as many relevant papers as possible. Where appropriate, controlled terms were exploded to retrieve records containing more specific terms and text words were searched both in singular and plural forms. The following MEDLINE search strategy was used: (oils, volatile [mh] OR “essential oil*” OR “volatile oil*” or “rapid evaporating oil*” OR “rapid evapourating oil*”) AND (herpesvirus 1, human [mh] OR “human alphaherpesvirus 1” OR “herpes simplex virus 1” OR “herpes simplex virus I” OR “herpes simplex virus type 1” OR “herpes simplex virus type I” OR “hsv1” OR “hsv 1” OR “herpes virus 1” OR “herpes virus I” OR “herpesvirus 1” OR “herpesvirus I” OR “herpes virus type 1” OR “herpes virus type I” OR “hhv1” OR “hhv 1” OR herpes labialis [mh] OR “herpes labialis” OR “herpes facialis” or “herpes simplex labialis” OR “labial herpes” OR “herpes”. Additional articles were searched manually based on the reference lists of relevant studies retrieved. *Data extraction*: Studies concerning the effectiveness of EOs against HSV‐1 were included. Contact with authors was not necessary. In presence of repeated articles, the most recent was considered. Table 1 summarizes the extracted data. *Assessment of biases:* Biases were identified as absence of statistical analysis, binomial nomenclature of aromatic plant, EO quality analysis, and quantitative data.

**Table 1:** Summary table of the results of the bibliographic search.

Author	Essential oil	Cell line	GC	Main components	Antiviral assay	Results	Statistical analysis	ρ	Bias	Main result
Monica Rosa Loizzo (2008) (Loizzo et al. 2008)	*C. libani A. Rich*. Essential oil	Vero cells ‐ Monkey kidney cell line	Yes	himachalol (22.50%), b‐himachalene (21.90%), and a‐himachalene (10.50%)	Cytopathic effect assay	IC50: 0,025‐0,8 µg/ml	/	/	(A)	/
Paul Schnitzler (2007)	*Zingiber officinale, Thymus vulgaris*, *Hyssopus officinalis*, and *Santalum album* essential oils	RC‐37	No	/	Plaque reduction assay	*Zingiber officinalis*: EC50:0,0002 ± 0.00001% *Thymus Vulgaris*: EC50 0.001 ± 0.0001% *Hyssopus Officinalis*: EC50 0.0001 ± 0.00001% *Santalum Album*: EC50 0.0002 ± 0.000003%	/	/	(A), (C)	*Thymus vulgaris*
P. Schnitzler (2007) (Schnitzler et al. 2007)	*Leptospermum scoparium*.	RC‐37	Yes	*Leptospermim scoparium*: leptospermone (14.36%), calamene (12,93%)	Plaque reduction assay	Plaque reduction: > 99%	/	/	(A)	*/*
Giulia Vanti (2020) (Vanti et al. 2020)	*Melissa officinalis* essential oil	Vero cells ‐ Monkey kidney cell line	Yes	Geranial (36.73%), Neral (27.31%), β‐ Caryophyllene (14.85%)	Luciferase assay	IC50: 4 μg/ml	/	/	(A)	
M. Heidary Navid (2013) (Heidary Navid et al. 2013)	Cajuput Oil	RC‐37	No	/	Attachment and penetration assay, plaque reduction assay	IC50: 7.5 μg/ml	/	/	(A), (B), (C)	
Valenti (2001) (Valenti et al. 2001)	*Santolina insularis* essential oil	Vero cells ‐ Monkey kidney cell line	No	/	Plaque reduction assay, post‐attachment assay, attachment assay, Yield reduction assay.	IC50: 0.88 μg/ml	/	/	(A), (C)	
Livia Civitelli (2014) (Civitelli et al. 2014)	*Mentha suaveolens* and *Melaleuca alternifolia*	Vero cells ‐ Monkey kidney cell line	Yes	Piperitone oxide (PEO) (80‐90%), limonene, alfa‐cubebene and pulegone	Plaque reduction assay	*Mentha Suaveolens*: IC50 at doses of 30 μg/ml 5.1 ± 0.48 μg/ml *Melaleuca Alternifolia*: IC50 at doses of 30 μg/ml 13.2 ± 0.21 μg/ml	Student's *t* test	< 0.05 and < 0.01	/	*Mentha Suaveolens*
Masato Minami (2003) (Minami et al. 2003)	*Cupressus sempervirens, Juniperus communis, Melaleuca alternifolia, Ocimum basilicum album, Mentha piperita, Origanum majorana, Eucalyptus globulus, Ravensara aromatica, Lavandula latifolia, Citrus limonum, Rosmarinus officinalis, Cymbopogon citratus*.	Vero cells ‐ Monkey kidney cell line	No	/	Plaque reduction assay	*Cypressus sempervirens*: at 1% the % of plaque formation was 42.8 ± 12.3 *Juniperus communis*: At 1% the % of plaque formation was 119 ± 4.1 *Melaleuca Alternifolia*: at 1% concentration it inhibited the growth of HSV‐1 *Ocinum basilicum album*: at 1% the % of plaque formation was 73.8 ± 4.1 *Mentha piperita*: at 1% concentration it inhibited the growth of HSV‐1 *Origanum majorana*:at 1% concentration it inhibited the growth of HSV‐1 *Eucalyptus globulus*: at 1% concentration it inhibited the growth of HSV‐1 *Ravensara aromatica*: at 1% concentration it inhibited the growth of HSV‐1 *Lavandula latifolia*: at 1% concentration it inhibited the growth of HSV‐1 *Citrus limonum*: at 1% concentration it inhibited the growth of HSV‐1 *Rosmarinus officinalis*: at 1% concentration it inhibited the growth of HSV‐1 *Cymbopogon citratus*: at 0.1% it inhibited the growth of HSV‐1	/	/	(A), (C)	*Cymbopogon citratus*
Christine Koch (2008) (Koch et al. 2008)	*Illicium verum, Pinus mugo, Matricaria recutita*	RC‐37, Vero cells and MCDK	No	/	Plaque reduction assay	*Illicium verum*: IC50 40 ± 4.0 μg/ml *Pinus mugo*: IC50 7 ± 0.84 μg/ml *Matricaria recutita*: IC50 0.3 ± 0.045 μg/ml	Mann–Whitney method	/	(C)	*Matricaria recutita*
J. Sharifi‐Rad (2017) (Sharifi‐Rad et al. 2017)	*Sinapis arvensis L., Lallemantia royleana Benth*. and *Pulicaria vulgaris Gaertn*	Vero cell line CCL‐81‐ATCC	No	/	Plaque reduction assay	*Sinapis arvensis* L.: IC50 0.035% *Lallemantia royleana Benth*: IC50 0.011% *Pulicaria vulgaris Gaertn*: IC50 0.001%	/	/	(A), (C)	*Pulicaria vulgaris Gaertn*
A. Schuhmacher (2003) (Schuhmacher et al. 2003)	*Mentha piperita*	RC‐37	Yes	menthol (42.8%), menthone (14.6%)	Plaque reduction assay	IC50: 0.002% Plaque reduction formation: 82% at a concentration of 0.01%	/	/	(A)	
F. Benencia (1999) (Benencia and Courrèges 1999)	Essential oil of *Santalum album L*.	Vero cell	No	/	Plaque reduction assay	EC50: 25 μg/ml	T‐student test	*p* < 0.01	/	
Akram Astani (2011) (Astani et al. 2011)	Star anise oil	RC‐37	No	/	Plaque reduction assay	IC50: 1 ± 0.1 μg/ml	t‐test	< 0.5	(B), (C)	
Farouk K. El‐Baz (2015) (El‐baz et al. 2015)	Leaf essential oil of Eucalyptus camaldulensis.	Vero cells	No	/	Plaque reduction assay	Nontoxic dilutions were mixed (100 µl) with 100 µl of different doses (1×10⁵, 1×10⁶, 1×10⁷ PFU/ml) of Herpes virus type 1: with all these different doses 90% reduction was observed using 1/10 dilution of 100 µL	/	/	(A), (C)	
P. Schnitzler (2008) (Schnitzler et al. 2008)	*Melissa officinalis*	RC‐37	Yes	Citral a (20.13%), Caryophyllen (17.31%), Citral b (13.58%)	Plaque reduction assay	At a concentration of 0.002% of Melissa Officinalis essential oil the titers were reduced by 98.8%	/	/	(A)	
Madson Ralide Fonseca Gomes (2012) (Fonseca Gomes et al. 2013)	Drimys angustifolia and D. brasiliensis	Vero cell line CCL‐81‐ATCC	Yes	D. angustifolia: bicyclogermacrene (19.7%); D. brasiliensis: cyclocolorenone (18.3%)	Viral titer reduction assay	*Drimys angustifolia*: 156.3 μg/mL *Drimys brasiliensis*: 625.0 μg/mL	/	/	(A)	*Drimys angustifolia*
Ilkay ERDOÿAN ORHAN (2012) (Erdoǧan Orhan et al. 2012)	*Anethum graveolens, Foeniculum vulgare* (fully mature), *Foeniculum vulgare* (flowering), *Lavandula officinalis, Mentha piperita, Mentha spicata, Ocimum basilicum* (green variety), *Ocimum basilicum* (purple variety), *Origanum onites, Origanum majorana, Origanum munitiflorum, Origanum vulgaris, Rosmarinus officinalis, Salvia officinalis, Satureja cuneifolia*	Vero cells	No	/	Antiviral Assay	*Anethum graveolens*: Cytopathogenic effect 0,025‐0,8 µg/ml *Foeniculum vulgare* (fully mature): Cytopathogenic effect 0,2‐0,4 µg/ml *Foeniculum vulgare* (flowering): Cytopathogenic effect: 0,8 µg/ml *Lavandula officinalis*: Cytopathogenic effect 0,8 µg/ml *Mentha piperita*: Cytopathogenic effect 0,2‐0,4 µg/ml *Mentha spicata*: Cytopathogenic effect 0,2 − 0,4 µg/ml *Ocimum basilicum* (green variety): Cytopathogenic effect 0,8 µg/ml *Ocimum basilicum* (purple variety): Cytopathogenic effect 0,8 µg/ml *Origanum onites*: Cytopathogenic effect 0,8 µg/ml *Origanum majorana:* Cytopatogenic effect 0.8 µg/ml *Origanum munitiflorum*: Cytopathogenic effect 0,2 − 0,4 µg/ml *Origanum vulgaris*: Cytopathogeni effect 0,2 − 0,4 µg/ml *Rosmarinus officinalis*: Cytopathogenic effect 0.8 µg/ml *Salvia officinalis:* Cytopathogenic effect 0,8 µg/ml *Satureja cuneifolia*: Cytopathogenic effect 0,2 − 0,4 µg/ml	/	/	(A), (C)	
Shahin Gavanji (2014) (Gavanji et al. 2014)	*Zataria multiflora, Eucalyptus caesia, Rosmarinus officinalis, Satureja hotensis, Artemisia kermanensis*	Vero cell	Yes	Z. Multiflora: p‐Cymene (11.34%), Thymol (33.05%), Carvacrol (25.88%) E. Caesia: p‐Cymene (14.11%), 1,8‐Cineol (40.18%), γ‐Terpinene (12.43%), R. Officinalis: α‐Pinene (23.93%), Camphor (10.97%), Verbenon (15.44%) S. Hotensis: γ‐Terpinene (31.96%), Carvacrol (32.38%) A. Kermanensis: α‐Thujone (13.83%), Camphore (10.23%)	Plaque reduction assay	*Zataria multiflora*: IC50 0.003% *Eucalyptus caesia*: IC50 0.004% *Rosmarinus officinalis*: IC50 0.006% *Satureja hotensis*: IC50: 0.008% *Artemisia kermanensis*:IC50 0.007%	/	/	(A)	
Zenab Aly Torky (2021) (Torky et al. 2021)	*Phlomis aurea* essential oil	African green monkey kidney cells ATCC‐81	Yes	α‐Pinene (22.96%), Limonene (6.26%), trans‐β‐Farnesene (11.36%), Germacrene D (51.56%)	viral plaque formation number reduction assay	IC50: 25 μg/mL	ANOVA and Tukey's post hoc test	< 0.05	/	/
Abd El‐Nasser G. El Gendy (2022) (El Gendy et al. 2022)	Acacia nilotica essential oils from bark and fruits	VERO‐E6 cells	yes	Stachene (48.34%), Caryophyllene oxide (19,11%)	MTT test	Antiviral effect %: 35.98 ± 1.31 (bark EO), 14.26 ± 0.54 (fruits EO)	ANOVA	< 0,05	/	/
Neli Vilhelmova‐Ilieva (2021) (Vilhelmova‐Ilieva et al. 2021)	Rosa damascena and Rosa alba L.	Madin‐Darby bovine kidney cells	yes	Geraniol, citronellol, nerol, linalool, farnesol, nonadecene	cytopathic effect inhibition (CPE) test and virucidal assay	R. damascena Mill. Oil: at 120 min of incubation ∆lg = 4.25 reduction of viral titer) R. alba L. oil: ∆lg 3.75 at 120 min of incubation	student's *t*‐test	< 0.05	/	*R. damascena* Mill. Oil

*Note*. BIAS: (A) Absence of statistical analysis ρ (B) Absence of binomial nomenclature (C) Absence of essential oil quality analysis GC (D) Absence of quantitative data

### 2.2 Clinical Strains, Growth Medium, Natural Products

Microbiological analyses were conducted on three *Candida albicans* strains: the commercial reference strain ATCC 24433, and two clinical isolates derived from mucosal swabs isolated at the “Dipartimento di Scienze di Laboratorio e Infettivologiche” of the Policlinico A. Gemelli University Hospital in Rome—Italy—one antifungal‐sensitive (CA‐FPG2S) and one resistant to Voriconazole and Fluconazole (CA‐FPG1R). All strains were cultivated on Sabouraud Agar (Thermo Fisher Scientific. 168 Third Avenue. Waltham, MA USA 02451) or Sensititre YeastOne set (Thermo Fisher Scientific. 168 Third Avenue. Waltham, MA USA 02451) at 37°C for 24 h. Fourteen EOs (*Mentha piperita*, *Thymus vulgaris*, *Hyssopus officinalis*, *Zingiber officinalis*, *Eucalyptus globulus*, *Cymbopogon citratus*, *Matricaria recutita*, *Eugenia caryophyllata*, *Melaleuca alternifolia*, *Cymbopogon martinii*, *Illicium verum*, *Cinnamomum camphora* 1–8 cineole, *Eucalyptus radiata*, *Melaleuca quinquenervia*) and two commercial products [Buccarom (BA) and Labiarom (LA)], kindly provided by Pranarôm (Avenue des Artisans, 37 − 7822 Ghislenghien ‐ Belgium), were included in the study. *Rosa mosqueta* vegetal oil (Avenue des Artisans, 37 − 7822 Ghislenghien ‐ Belgium) was used as vehicle for the EOs selected to obtain the lip‐lotion. These products were evaluated both individually and in combination with the goal of formulating an EO‐based mixture.

### 2.3 Evaluation of Antifungal Susceptibility

Antifungal susceptibility testing was performed following CLSI guidelines (Clinical and Laboratory Standards Institute CLSI 2017) using the Sensititre YeastOne system (Thermo Fisher Scientific, 168 Third Avenue, Waltham, MA, USA 02451), a method commonly used in clinical routine. This test, based on a 96‐well plate format, was used to determine the Minimum Inhibitory Concentration (MIC) of the following antifungal agents: 5‐Flucytosine, Amphotericin B, Anidulafungin, Caspofungin, Fluconazole, Isavuconazole, Itraconazole, Micafungin, Posaconazole, Rezafungin, and Voriconazole. Briefly, for each strain a sterile water suspension with an optical density of 0.5 McFarlan were prepared. Subsequently, 20 μL of this suspension were diluted in yeast broth medium (Y3462, Thermo Scientific), and 100 μL was inoculated into each well.

For each of strain, a plate pretreated with the antifungal agents and alamarBlue Cell Viability Reagent (Invitrogen by Thermo Fisher Scientific, Eugene USA), required to assess the growth of the microorganisms, was prepared. Plates were incubated overnight at 37°C for 24 h. Subsequently, to identify the susceptibility to antifungal drug MIC values were detected (Pfaller et al. 1998).

### 2.4 Broth Micro‐Dilution Susceptibility Test

To evaluate the anti‐fungal effectiveness of natural compounds, the broth micro‐dilution susceptibility test as established by the European Committee on Antimicrobial Susceptibility Testing (EUCAST EDef 7.2 and EUCAST 5.0) (Arendrup et al. 2012) international guidelines was followed. Briefly, scalar dilutions ranging from 2% v/v to 0.015% v/v or 4% v/v and 0.03% v/v were tested for EOs alone or formulations, respectively. An equal concentration of Tween 80 (Sigma Aldrich, Saint Louis, MO, USA) was used as surfactant. A suspension of each strain was performed to obtain an optical density of 0.5 McFarland. The suspension was adjusted to have a final concentration equal to 0.5*10^5^ cfu/mL. 96 well‐plates were used for the test, and an overnight incubation at 37°C was performed. The MIC values were determined as the lowest EO concentration corresponding to the complete inhibition of fungal growth. Additionally, the Minimum Fungicidal Concentration (MFC), defined as the lowest EO concentration resulting in the absence of 99% of fungal growth, was determined by plating 5 μL of the contents of each well on Sabouraud Agar plates and incubating them at 37°C for 24 h. Each experiment was in triple, and for each assay, a positive control lacking essential oil was included, alongside a negative control consisting exclusively of sterile culture medium to confirm the absence of contamination (Di Vito et al. 2022).

### 2.5 Essential Oils (EOs) Formulation

Based on the results obtained from the broth micro‐dilution susceptibility tests, four EOs with the best antifungal activity were selected to obtain a lip formulation (MIX) with a final concentration of 2% v/v. *M. piperita* (0.4% v/v), *E. caryophyllata* (0.4% v/v), *C. martinii* (0.4% v/v), and *C. camphora* chemotype 1,8‐cineole (0.8% v/v) were included. EOs selected for the formulation, in addition to their antifungal activity, were chosen for their anti‐inflammatory and anti‐pain (*M. piperita*, *E. caryophyllata*), anti‐microbial (*C. martinii*) and anti‐ viral (*C. camphora*) activity. *T. vulgaris* was excluded to avoid excessive phenolic components and to maintain the overall sensory acceptability of a formulation deemed balanced. The latter, due to its traditional antiviral use, was included in double concentration compared to the others. *Rosa mosqueta* vegetal oil was used as vehicle (Pranarôm, Avenue des Artisans, 37 − 7822 Ghislenghien ‐ Belgium).

### 2.6 Solid Phase Microextraction (SPME) Sampling

SPME, a headspace sampling technique, was employed to describe the volatile composition of all EOs and formulations. Briefly, a small amount of each sample was individually inserted in a specially designed vial and sealed with a perforated PTFE cap. The selected adsorption fiber was coated with 50/30 μm DVB/CAR/PDMS (divinylbenzene/carboxen/polydimethylsiloxane). After approximately 2 min of sampling at an optimized temperature of around 60°C, the fiber was inserted into the GC injector maintained at 250°C to release the captured analytes (Rizzo et al. 2023).

### 2.7 GC‐MS Analysis

Chromatographic analyses of the EOs, the EO mix, and LA commercial formulation were carried out by gas chromatograph coupled with mass spectrometer detector (Clarus 500 model, Perkin Elmer). The GC was equipped with a Flame Ionization Detector (FID) for relative quantitative analyte determination. An apolar capillary column (Varian Factor Four VF‐1) was employed to separate the compounds and hyper pure Helium serving as the carrier gas at a constant flow rate of 1.0 mL/min. The temperature program included a ramp from 60°C to 220°C at a rate of 6°C per minute, followed by a constant temperature of 220°C for 20 min. The mass spectra were obtained in the electron impact mode (EI) at 70 eV in scan mode in the range 35–450 m/z. Compound identification was achieved through comparison with mass spectra available in database Nist11 and by comparing the calculated linear retention indices (LRIs), with those reported in the literature. Relative concentrations, expressed as percentages, were determined by calculating the ratio of the peak area of each individual compound to the total peak area. All analyses were performed in triplicate for robustness (Rizzo et al. 2023).

### 2.8 Basophil Activation Test (BAT)

From April to July 2022, the allergenicity of MIX and LA was assessed using the BAT, following the protocol described by Hoffmann Hj in 2015 (Arendrup et al. 2012), with the Flow CAST kit (FK‐CCR‐U, BÜHLMANN Diagnostics Corp). The protocol includes the use of fluorescently labeled monoclonal antibodies (anti‐CD63‐FITCH and anti‐CCR3‐PE) for staining human CD63 and CCR3 molecules. Flow cytofluorimetry (Sainte‐Laudy et al. 1994) was used for visualization. The EOs of *M. piperita*, *E. caryophyllata*, *C. martinii, C. camphora* 1,8‐cineole, MIX and LA were used as allergen. Briefly, for each EO a mother suspension made 20 times more concentrated than the Minimum Inhibitory Concentration (MIC) with an equal amount of surfactant (Tween 80, Sigma Aldrich) was prepared. 500 μL of stimulation buffer were used to have mother solutions at the following concentrations: 5% for *M. piperita*, 1.2% *E. caryophyllata*, 2.5% *C. martinii*, 20% *C. camphora* 1,8‐cineole 20%. Lip Cream was tested at a 1:2 dilution in stimulation buffer and formulated as an 8% v/v solution. A 1:10 dilution of the same mother used for microbiological tests was used as the stock suspension for MIX. 1:100, 1:1,000, and 1:10,000 dilutions of stock suspension were tested. Fresh blood samples from 10 donors volunteers (five volunteers diagnosed with allergies and five healthy volunteers) were used. Each analyzed sample contained: 100 μL of stimulation buffer, 50 μL of allergen or positive control or stimulation buffer (negative control), 50 μL of whole blood, and 20 μL of staining reagent. Samples were incubated for 15 min at 37°C. After this period, 2 mL of pre‐heated lysis reagent (18°C–28°C) was added, followed by 10‐min incubation, centrifugation and discarding of the supernatant. 300 μL of wash buffer was added to each sample and flow cytometric reading was done. Flow cytometry software was used to set a gate around CCR3‐positive basophils with low side scatter (SSC) to calculate the percentage of CD63‐positive cells relative to total basophil count. “Positive” was considered a sample with a percentage of activated basophil higher than 5% in almost two of the three test performed for each natural substances.

### 2.9 Peripheral Blood Mononuclear Cell (PBMC) Collection and ELISA Test

To study the anti‐inflammatory activity of formulations, PBMCs were collected from a pool of 22 fresh blood samples of healthy donors volunteers using HISTOPAQUE‐1077 (Sigma Aldrich, St. Louis, MO, USA) as the manufacturer's instructions and already described in a previous publication (Di Vito et al. 2020). 1 × 10^6^ cells/mL suspension was cultured in octuplicate using a 24‐well cell culture plate at 37°C for 24 h. PBMCs were treated with lipopolysaccharide (LPS, 1% v/v starting from a stock solution of 1 µg/mL) alone or in combination with LA or MIX suspensions (dilution equal to 1:1.000) prepared as described in paragraph “Basophil activation test” (using PBS instead of stimulation buffer). Three out of nine replicates were treated with 5 µL of Alamar Blue reagent (Invitrogen, Carlsbad, CA, USA) to assess cellular viability. Enzyme‐linked immunosorbent assays (ELISA) (Arigo biolaboratories, 22, Lane 227 Gongyuan Road Hsinchu City 300 Taiwan) were employed to measure the expression of IL‐6, INF gamma, TNF‐α, and IL‐1 cytokines. Cytokine content in each well was determined by optical reading at 450 nm using a microplate reader. Experiments included a calibration curve with standards at high concentration, medium and low concentration.

### 2.10 Cell Culture

To validate the *in vitro* anti‐viral activity of both MIX and LA, renal epithelial cell line VERO E6 monkey (ATCC No. CRL‐1586) was used. Cells were cultured in Minimum Essential Medium (MEM with l‐Glutamine, Gibco, Life Technologies Europe UK) supplemented with 10% Fetal Bovine Serum (FBS Gibco Origin Brazil, UK), 1% penicillin and streptomycin (P/S, Gibco, Life Technologies Corporation, NY USA), and 1% amphotericin (Amphotericin B 100X Sterile Filtered Euroclone S.p.A Pero, MI, Italy). Cells were seeded in 24‐well or 96‐well plates to form monolayers at 37°C with 5% CO_2_ overnight.

### 2.11 *In Vitro* Toxicity Test

*In vitro* toxicity test was done in a 96‐well flat‐bottom plate. To form a monolayer, 100 μL of 100,000‐cell suspension was incubated overnight at 37°C with 5% CO_2_. In each experiment, 100 µL of several concentrations of LA (0.16%, 0.08%, 0.008% v/v) and MIX (0.05%, 0.005%, 0.002%, 0.001%, 0.0005% v/v) mixed with 0.5% v/v Tween 80 surfactant were used. Treated samples were incubated at 37°C for 2 h, then they were washed, fresh culture medium was added to incubate samples for other 3 days. Alamar Blue Cell Viability Reagent (1:10 dilution) was added to each well, and the plate was incubated at 37°C until color change detected using a spectrophotometer (560 nm and 605 nm wavelengths). Positive (cells infected with HSV‐1) and negative controls (cells in culture medium only) were included in each experiment carried out in triplicate (Kumar et al. 2018).

### 2.12 *In Vitro* Antiviral Efficacy Test

To test the antiviral activity of LA and MIX, 2.5 × 104 cells Vero cells at 90% confluence were seeded in each well of a 24‐well plate and incubated overnight at 37.5°C with 5% CO_2_ to form a monolayer. Mother solutions made as described in paragraph “Basophil activation test” were diluted to test final concentrations equal to 0.16%, 0.08%, and 0.01% v/v of LA and 0.001%, 0.0005%, and 0.00005% v/v of MIX in cell culture medium containing 2% FBS and no antibiotics or antifungals. 10^4^ pfu/mL of HSV1 suspension with 2% of FBS was used to treat the monolayer for 1.5 h at 37°C and 5% CO_2_. After the cell monolayer was established, the cells were subjected to four distinct treatments using a final viral concentration of 10^4^ PFU/mL. Specifically: (i) cells were pre‐treated for 2 h with one of the LA or MIX dilutions prior to viral inoculation (aimed at assessing the protective effect of the mixtures on the cells); (ii) the viral suspension was first incubated with LA or MIX dilutions for 2 h at 37°C, and the resulting mixture was then used for infection (aimed at evaluating the impact on viral structure); (iii) cells were exposed simultaneously to LA or MIX dilutions and the viral suspension for 2 h at 37°C (aimed at determining efficacy during the viral entry phase); and (iv) cells were initially infected, for 2 h at 37°C, with the viral suspension and subsequently treated with LA or MIX dilutions (aimed at assessing efficacy during the post‐infection phase). At the end of each incubation time, the treatment was removed, the monolayer was washed and covered with soft gel agar made with 250 µL of cultured medium with 0.5% of Top Vision Low Melting Point Agarose (Thermo Fisher Scientific, Vilnus Lithuania, Spain) and 2% of FBS to wait plaques formation. Plates were incubated at 37°C for 3 days before staining the monolayer with crystal violet for 15 min. Plaques were counted using Cytation 5 (BioTek) with the Gene5 program. Results were compared infected controls, as well as untreated and uninfected negative controls. Each experiment was repeated in triplicate including an untreated control (positive control) and an uninfected control (negative control) (Yin et al. 2023).

### 2.13 Statistical Analysis

In accordance with a normal distribution of continuous variables, data were analyzed considering tests based on the mean and standard deviation values. Data obtained from microbiological and ELISA tests were represented as means ± standard deviations. As specified in paragraphs relating to each method, all data were obtained at least in triplicate and the results were defined as statistically significant for *p* < 0.05. The statistical analysis relating to the activity of LA and MIX against HSV‐1 was performed using Ordinary one‐way ANOVA test. For data processing the software GraphPad Prism v.8 was used (GraphPad Software Inc., San Diego, CA, USA).

## 3 Results

### 3.1 Bibliographic Search

**Figure 1:**
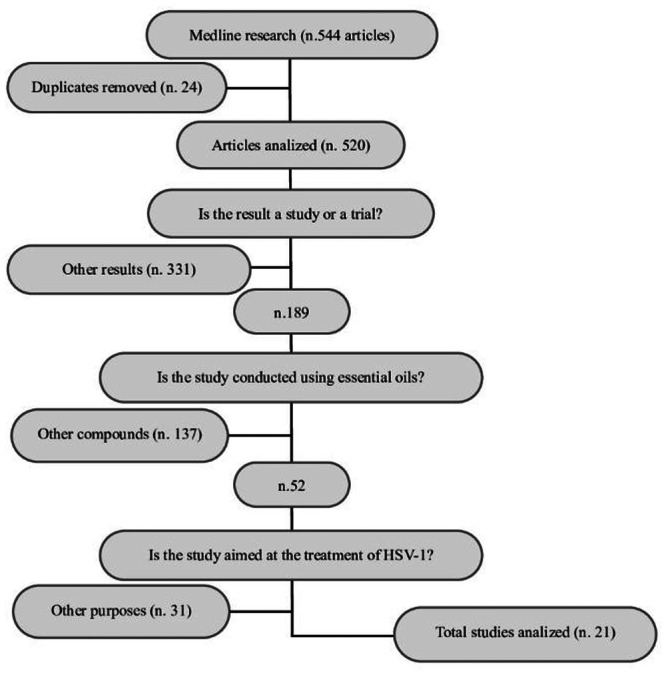
Flowchart of the study.

Figure 1 represents the flowchart of the study. 544 articles were identified by Medline and Embase, 523 studies were excluded (24 duplicates, 331 other results, 137 articles concerning other natural compounds, 31 other purposes). Table 1 reports data obtained from the bibliographic research. Specifically, the following information was extrapolated: (a) the author and the publication year, (b) EOs, (c) cell line, (d) the presence of EO quality analysis, (e) main compounds, (f) antiviral assay, (g) results, (h) presence of statistical analysis, (i) statistical power, (j) biases (k) main results. 21 in vitro studies were eligible; eleven of these evaluated the effectiveness of a single EO, while the others studied mixtures of EOs. Ten out of 21 articles did not show quality analysis and only 7 out of 21 articles showed statistical analysis.

11 out of 21 articles evaluated the effectiveness of a single essential oil, while the others studied mixtures of EOs. 10 out of 21 articles did not present gas chromatographic analysis, and only 4 showed statistical analysis. The bibliographic search shows that EOs active against HSV‐1 are obtained from *Thymus vulgaris*, *Mentha piperita*, *Matricaria recutita*, *Hyssopus officinalis*, *Eucalyptus globulus*, *Cymbopogon citratus* and *Zingiber officinalis*. These essential oils were selected for the investigation conducted in this study.

### 3.2 Evaluation of Antifungal Susceptibility

MIC values of *C. albicans* strains towards common antifungals analyzed in clinical practice are reported in Table 2. Data show that both the reference strain (*C. albicans* ATCC 24433) and the susceptible ones (*C. albicans* CA‐FPG2 S) were sensitive to all drugs, while the resistant clinical strain (*C. albicans* CA‐FPG1 R) showed resistance to voriconazole and fluconazole.

**Table 2:** MIC values of *C. albicans* strains towards common antifungals analyzed in clinical practice.

MIC			
	*C. albicans* ATCC 24433	*C. albicans* CA‐FPG1 R	*C. albicans* CA‐FPG2 S
Fungal anidula	0,015	0,015	0,015
Amphotericin B	0,5	0,5	0,5
Micafungin	0,015	0,015	0,008
Caspofungin	0,015	0,03	0,03
5‐Flucytosine	0,12	4	0,06
Posaconazole	0,06	0,5	0,03
Voriconazole	0,015	8	0,008
Itraconazole	0,12	0,5	0,06
Fluconazole	0,5	256	0,25

### 3.3 Broth Micro‐Dilution Susceptibility Test

MIC and MFC values of the 15 essential oils and the 2 commercial products tested against the 3 strains of *C. albicans* are shown in Table 3, respectively. EOs that showed greater effectiveness were *M. piperita* (MIC_Average _= 0.41 ± 0.09%v/v, MFC_Average_ = 0.51 ± 0.25%v/v), *T. vulgaris* (MIC_Average_ = 0.38 ± 0.10%v/v, MFC_Average_ = 0.50 ± 0.10%v/v), *E. caryophyllata* (MIC_Average_ = 0.06 ± 0.01%v/v, MFC_Average_ = 0.09 ± 0.02%v/v) and *C. martini* (MIC_Average_ = 0.125 ± 0.00%v/v, MFC_Average_ = 0.16 ± 0.08%v/v). Three of these EOs (*M. piperita, E. caryophyllata*, and *C. martini)* were mixed together with *C. camphora*, an EO with minor antifungal activity but traditionally used as an anti‐viral, in a ratio of 1:1:1:2. This formulation (MIX) shows the following antifungal values: MIC_Average_ = 0.23 ± 0.03%v/v, MFC_Average_ = 0.30 ± 0.07%v/v. Of the two commercial products, the one with greater effectiveness is the LA which showed average MIC values of 3.6 ± 0.58%v/v. Whereas it was not possible to identify the MFC values as, at the concentrations tested, LA showed only an inhibitory activity. Finally, MIX showed MIC and MFC average values of 0.23 ± 0.03%v/v and 0.30 ± 0.07%v/v respectively. Considering that EOs content in the LA commercial formulation is equal to 6%, the concentration of EOs at the MIC value corresponds to about the 0.26% v/v. The data show that both LA and MIX have the same inhibitory activity against the tested fungal strains.

**Table 3:** MIC and MFC values of natural products alone or in combination against *C. albicans* strains.

	MIC (% v/v)			MFC (% v/v)		
	*C. albicans* ATCC 24433	*C. albicans* CA‐FPG1 R 1	*C. albicans* CA‐FPG2 S 2	*C. albicans* ATCC 24433	*C. albicans* CA‐FPG1 R	*C. albicans* CA‐FPG2 S
*M. piperita*	0.33 ± 0.04	0.40 ± 0.00	0.50 ± 0.07	0.75 ± 0.43	0.25 ± 0.2	0.54 ± 0.20
*T. vulgaris*	0.50 ± 0.00	0.30 ± 0.11	0.35 ± 0.16	0.62 ± 0.33	0.45 ± 0.19	0.45 ± 0.26
*H. officinalis*	2.04 ± 0.8	1.83 ± 0.29	1.33 ± 0.58	2.04 ± 0.79	2.16 ± 0.28	1.83 ± 0.28
*Z. officinalis*	4.00 ± 0.00	4.00 ± 0.00	4.00 ± 0.00	> 4.00 ± 0.00	> 4.00 ± 0.00	> 4,00 ± 0.00
*E. globulus*	4.00 ± 0.00	2.33 ± 0.60	3.33 ± 0.60	> 4.00 ± 0.00	3.00 ± 1.00	3.33 ± 0.57
*C. citratus*	4.00 ± 0.00	2.33 ± 0.60	3.33 ± 0.60	0.2 ± 0.03	0.18 ± 0.00	0.23 ± 0.07
*M. recutita*	> 4 ± 0.00	> 4 ± 0.00	> 4 ± 0.00	> 4.00 ± 0.00	> 4.,00 ± 0.00	> 4.00 ± 0.00
*E. cariophyllus*	0.06 ± 0.00	0.06 ± 0.00	0.05 ± 0.00	0.09 ± 0.00	0.10 ± 0.02	0.07 ± 0.02
*M. alternifolia*	1.5 ± 0.00	1.5 ± 0.00	1.5 ± 0.00	4.00 ± 0.00	4.00 ± 0.00	4.00 ± 0.00
*C. martini*	4.00 ± 0.00	2.33 ± 0.60	3.33 ± 0.60	0.2 ± 0.03	0.18 ± 0.00	0.23 ± 0.07
*I. verum*	> 4 ± 0.00	> 4 ± 0.00	> 4 ± 0.00	> 4.00 ± 0.00	> 4.00 ± 0.00	> 4.00 ± 0.00
*C. camphora* (1.8‐cineolo)	0.05 ± 0.00	0.06 ± 0.00	0.05 ± 0.00	0.09 ± 0.00	0.10 ± 0.02	0.07 ± 0.02
*E. radiata*	1.5 ± 0.00	1.5 ± 0.00	1.5 ± 0.00	4.00 ± 0.00	4.00 ± 0.00	4.00 ± 0.00
*C. limon*	0.12 ± 0.00	0.12 ± 0.00	0.125 ± 0.00	0.25 ± 0.08	0.12 ± 0.00	0.12 ± 0.00
*M. quinquenervia*	3.00 ± 0.00	3.00 ± 0.00	1.50 ± 0.00	> 4.00 ± 0.00	> 4.00 ± 0.00	> 4.00 ± 0.00
Labial gel	1.00 ± 0.00	1.00 ± 0.00	1.00 ± 0.00	4.00 ± 0.00	4.00 ± 0.00	2.00 ± 0.00
Essential oils mix	4.00 ± 0.00	4.00 ± 0.00	4.00 ± 0.00	> 4.00 ± 0.00	> 4.00 ± 0.00	> 4.00 ± 0.00

^1^ Sensible clinical strain.

^2^ Resistant clinical strain.

### 3.4 GC‐MS Analyses

To evaluate the volatile chemical composition of both the EOs found to be most active against fungal cells and lip formulations (LA and MIX), GC/MS analyses were performed. Data in Table 4 showed that the major compounds with relative concentrations greater than 5% in *C. camphora* (1,8‐cineole) EO are: 1,8‐cineole (68.2%) and α‐terpineol (9.6%), and sabinene (7.4%). *E. caryophyllata* EO is characterized by 3 major compounds: eugenol (81.1%), eugenol acetate (11.5%) and β‐caryophyllene (5.5%). *C. martinii* EO is constituted by 2 main components: cis‐geraniol (83.1%) and geranyl acetate (10.9%). In *Mentha piperita* EO levomenthol (50.2%), I‐menthone (26.2%), menthol acetate (8.9%) and 1,8‐cineole (6.7%) were the principal molecules. Finally, LA consists essentially of cis‐sabinene hydrate (72.9%), while MIX shows 4 main components: 1,8‐cineole (28.4%), eugenol (19.1%), nerol (17.6%) and menthol (6%).

**Table 4:** Chemical composition of EOs, BA, LA, and MIX (percentage mean values).

N°	Component^a^	LRI ^b^	LRI ^c^	*C. camphora* (%)	*E. caryophyllata (%)*	*C. martinii (%)*	*M. piperita (%)*	Labial gel (%)	Mix in Rose hip oil (%)
1	α‐thujene	821	823	—	—	—	tr	0.6	0.3
2	α‐pinene	941	942	2.3	—	—	0.4	3.8	1.9
3	3‐methylcyclohexanone	943	945	—	—	—	tr	—	—
4	camphene	945	946	tr	—	—	tr	—	—
5	β‐thujene	971	968	0.4	—	—	—	—	—
6	sabinene	973	972	7.4	—	—	0.2	—	—
7	β‐pinene	979	978	3.2	—	—	0.8	5.0	—
8	β‐myrcene	985	987	—	—	0.4	—	—	0.6
9	α‐phellandrene	1002	1006	—	—	—	—	0.2	—
10	p‐cymene	1012	1016	—	—	—	0.2	0.7	—
11	1,8‐cineole	1030	1033	68.2	—	0.1	6.7	—	28.4
12	β‐terpinene	1033	1036	—	—	—	—	—	1.5
13	cis‐β‐ocimene	1034	1035	—	—	—	—	—	0.5
14	limonene	1038	1037	—	—	tr	—	—	—
15	γ‐terpinene	1051	1048	1.5		—	0.2	3.1	1.0
16	trans‐β‐ocimene	1060	1059	—	—	0.1	tr	—	—
1	cis‐sabinene hydrate	1068	1069	tr		—	—	72.9	—
18	terpinolene	1089	1092	0.4	—	1.8	tr	3.2	—
19	trans‐sabinene hydrate	1099	1098	0.2	—	—	tr	0.1	—
20	1,3,8‐p‐menthatriene	1120	1118	—	—	—	—	—	0.6
21	camphor	1130	1126	—	—	—	—	0.1	—
22	bilagen	1133	1132	—	—	—	—	—	0.3
23	p‐menthone	1137	1135	—	—	—	—	—	3.8
24	I‐menthone	1145	1142	—	—	—	26.2	1.2	0.5
25	levomenthol	1155	1150	—	—	—	50.2	0.2	0.4
26	menthol	1156	1153	—	—	—	—	1.3	6.0
27	terpinene‐4‐ol	1161	1160	3.2	—	—	—	—	1.6
28	methyl salicylate	1168	1166	—	0.1	—	—	—	—
29	α‐terpineol	1190	1183	9.6	—	—	—	3.6	2.7
30	linalool formate	1210	1206	—	—	0.9	0.2	—	—
31	carveol	1219	1217	—	—	—	—	0.1	—
32	β‐citral	1221	1219	—	—	—	—	0.2	—
33	pulegone	1225	1220	—	—	—	0.9	—	—
34	nerol	1230	1227	—	—	—	—	—	17.6
35	piperitone	1235	1231	—	—	—	—	—	—
36	cis‐geraniol	1237	1236	0.1	—	83.1	—	—	—
37	chavicol	1250	1254	—	0.1	—	—	—	—
38	lavandulol acetate	1268	1271	—	—	0.2	—	—	4.0
39	menthol, acetate	1298	1294	—	—	—	8.9	0.3	—
40	menthyl acetate	1308	1304	—	—	—	0.4	—	—
41	eugenol	1350	1345	—	81.1	—	—	—	19.1
42	nerol acetate	1366	1363	—	—	0.2	—	—	—
43	geranyl acetate	1368	1366	—	—	10.9	—	—	—
44	α‐copaene	1370	1368	—	—	—	0.1	—	—
45	α‐bourbonene	1376	1374	tr	—	—	—	—	—
46	isogermacrene D	1389	1385	—	—	—	tr	—	—
47	(‐)‐β‐bourbonene	1395	1390	—	—	—	0.2	—	—
48	α‐copaene	1396	1392	tr	—	—	—	—	—
49	β‐elemene	1410	1406	0.2	—	—	—	—	—
50	β‐cubebene	1422	1420	—	—	—	0.4	—	—
51	γ‐elemene	1426	1423	—	—	—	0.1	—	—
52	β‐caryophyllene	1427	1424	0.8	5.5	1.6	2.4	0.7	2.7
53	β‐copaene	1430	1427	tr	—	—	—	—	—
54	isoeugenol	1441	1439	—	—	—	—	—	1.9
55	cis‐β‐farnesene	1453	1451	—	—	0.1	—	—	—
56	aromadendrene	1462	1460	0.1	—	—	—	0.1	—
57	humulene	1468	1465	1.0	1.2	0.2	0.1	0.2	—
58	chamigren	1479	1475	tr	—	—	—	—	—
59	γ‐gurjunene	1482	1479	0.1	—	—	—	—	—
60	β‐eudesmene	1484	1481	0.2	—	—	tr	—	—
61	eugenol acetate	1486	1482	—	11.5	—	—	—	—
62	elixene	1495	1492	0.1	—	—	—	—	—
63	viridiflorene	1499	1496	—	—	—	—	0.2	—
64	germacrene D	1505	1500	tr	—	—	0.1	—	—
65	α‐farnesene	1510	1507	tr	tr	—	—	—	—
66	geranyl isobutyrate	1512	1514	—	—	0.1	—	—	—
67	δ‐cadinene	1533	1530	tr	0.1	—	0.1	tr	—
68	nerolidol	1568	1565	—	—	tr	—	—	—
69	spathulenol	1573	1571	tr	—	—	—	—	—
70	viridiflorol	1579	1580	—	—	—	—	0.1	—
71	humulene epoxide II	1600	1605	tr	0.1	—	—	—	—
72	caryophyllene oxide	1620	1613	—	0.2	tr	—	—	—
73	farnesol	1664	1658	—	—	tr	—	—	—
74	β‐terpinyl acetate	1690	1684	—	—	—	—	0.3	—
	**SUM**			99.0	99.9	99.7	98.8	98.2	95.4

^a^ The components are reported according to their elution order on apolar column.

^b^ Linear Retention indices measured on apolar column.

^c^ Linear Retention indices from literature; tr: percentage mean values < 0.1; ‐: not detected.

### 3.5 Basophil Activation Test (BAT)

Table 5 shows results obtained by testing the individual EOs, MIX and LA against basophil cells. Only one sample showed activation greater than 5% in at least two of the three tests performed with the *C. camphora* EO. Generally, in the clinic this frequency of positive reaction identifies a positive test sample implying a possible systemic reaction. All other tests were negative except for C2 and C10 for which it was not possible to test the activity on *E. caryophyllata* EO due to widespread cell death. Therefore, except for one sample, none of the EOs or compounds tested showed basophil‐activating action.

**Table 5:** BAT analysis.

	Samples		Non allergic volunteers					Allergic volunteers				
			V1	V2	V3	V4	V5	V6	V7	V8	V9	V10
1	Control	Negative	0%	1%	0%	0%	0%	1%	0%	0%	1%	0%
		Positive	96%	93%	89%	81%	93%	59%	91%	84%	51%	97%
2	*E. caryophyllata*	1:100	0%	cell death	0%	1%	0%	0%	1%	0%	1%	cell death
		1:1000	0%	cell death	1%	1%	0%	0%	0%	0%	0%	cell death
		1:10000	0%	cell death	1%	0%	0%	0%	1%	0%	1%	cell death
3	*C. martinii*	1:100	1%	1%	1%	0%	1%	0%	0%	0%	1%	1%
		1:1000	0%	1%	0%	0%	0%	0%	0%	0%	1%	1%
		1:10000	0%	0%	1%	0%	0%	0%	1%	1%	0%	1%
4	*M. piperita*	1:100	1%	0%	0%	0%	1%	1%	0%	0%	0%	1%
		1:1000	0%	0%	0%	0%	1%	0%	0%	0%	0%	0%
		1:10000	0%	0%	0%	2%	0%	0%	0%	0%	1%	0%
5	*C. camphora CT 1‐8 cineole*	1:100	2%	1%	1%	0%	3%	2%	0%	0%	**8%**	1%
		1:1000	0%	1%	0%	0%	0%	0%	1%	0%	**6%**	0%
		1:10000	0%	0%	0%	0%	0%	0%	0%	0%	**4%**	1%
6	*MIX‐EO*	1:100	0%	1%	0%	0%	0%	1%	0%	0%	1%	1%
		1:1000	1%	1%	0%	0%	1%	0%	0%	0%	1%	0%
		1:10000	0%	1%	0%	1%	0%	1%	0%	0%	1%	1%
7	*MIX‐C*	1:100	0%	2%	0%	0%	0%	1%	1%	0%	0%	1%
		1:1000	1%	1%	0%	1%	0%	1%	0%	0%	1%	1%
		1:10000	0%	1%	0%	0%	0%	0%	1%	0%	0%	0%

### 3.6 Cytotoxicity Test

At the tested concentration of 1:1000, neither the commercial nor the MIX formulations showed significant cytotoxic effects on lymphomonocytes. This confirms that the selected concentration is safe for use in subsequent assays.

### 3.7 ELISA Test

**Figure 2:**
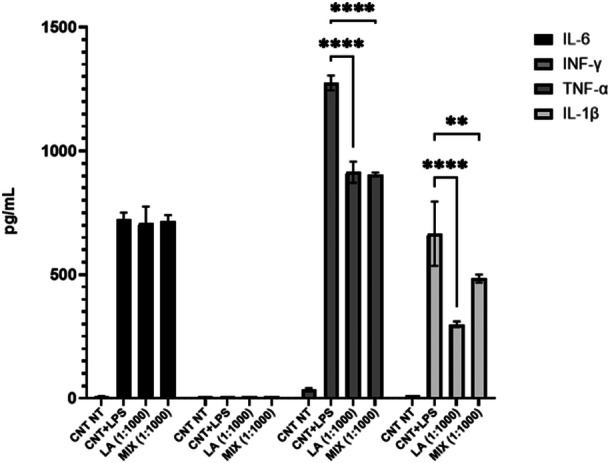
Expression of cytokines IL‐6, INF‐γ, TNF‐α and IL‐1β in presence of LPS, LA or MIX *stimuli*. CNT NT: untreated control, LPS; lipopolysaccharide. LA and MIX were tested at a 1:1000 dilution. ****p < 0.0001, **p < 0.001.

To evaluate the pro‐inflammatory activity of LA or MIX on the expression of IL‐6, INF‐g, TNF‐a, and IL‐1β cytokines, PBMC were incubated in culture medium with a 1:1000 dilution of the formulations. The dilution tested was first selected as non‐toxic for PBMC. As shown in Figure 2 the expression of all cytokines increases in presence of a stimulus. Specifically, the expression of TNF‐α (*p* < 0.0001) and IL‐1β (*p* = 0.0010) is lower and statistically significant in samples treated with LA (TNF‐α = −29.7% and IL‐1β = −50.0%) or MIX (TNF‐α = −33.6% and IL‐1β = −25.0%) if compared to LPS alone. This result indicates that LA and MIX stimulate these two pro‐inflammatory cytokines less than a stimulus (LPS) generally accepted as pro‐inflammatory.

### 3.8 *In Vitro* Antiviral Efficacy Test

**Figure 3:**
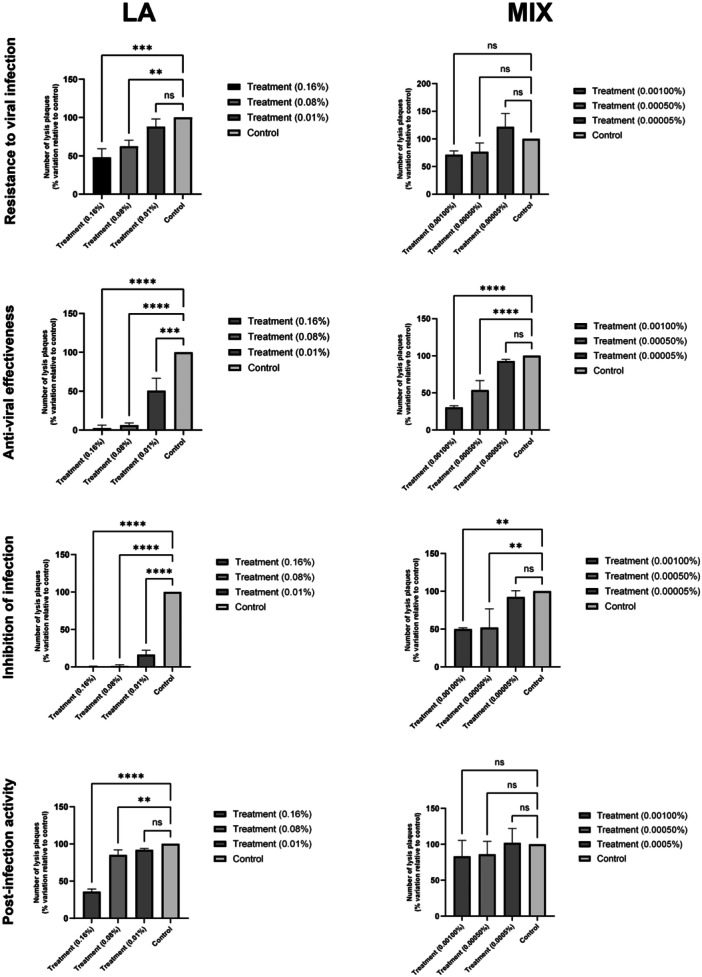
*In vitro* tests of anti‐HSV1 of LA and MIX. All concentrations tested are expressed as % v/v. ****p < 0.0001, ***p < 0.0002, **p < 0.005, ns = not statistically significant.

Figure 3 shows the results of the *in vitro* tests done to evaluate the anti‐HSV‐1 efficacy of MIX and LA. Data show that Vero cells, after pre‐treatment with the two maximum concentrations tested of both formulations (0.16% and 0.08% v/v for LA and 0.001% and 0.0005% for MIX), are more resistant to HSV‐1 infection. Formulations have also shown virucidal activity. In fact, the pre‐incubation of the HSV‐1 induces a significant dose‐dependent decrease (*p* < 0.0001) of the plaques number both for all three tested concentrations of LA (decrease equal to 97.5%, 93.8% and 49.4%) and for the two highest concentrations of MIX (decrease equal to 70% and 46.4%; *p* < 0.005). Formulations are also active during the viral infectious process. In fact, the incubation during infection shows a significant reduction (*p* < 0.0001) of the plaques number for all tested concentrations of LA (decrease equal to 99.5%, 98.8% and 83.7%), and for the two highest concentrations of MIX (decrease equal to 50% and 48% *p* < 0.005). Finally, while MIX is not active in inhibiting intra‐cellular viral replication, while LA shows significant dose‐dependent efficacy with adecrease equal to 64% (*p* < 0.0001), 15% (*p* < 0.005) and 8%.

## 4 Discussion

### 4.1 Literature Search

The literature analysis reveals a lack of thorough investigation into the effectiveness of EOs against HSV‐1. Only 21 articles were found, mostly in vitro studies with limitations such as absence of chemical quality analysis of EOs and incomplete species identification. Moreover, only a few studies include statistical data analysis, essential for validating scientific findings and ensuring safety. Unfortunately, the two clinical studies founded during the research lacked relevant data. One focused on patient perception rather than treatment effectiveness, while the other emphasized post‐treatment pain, healing time, and itching. Therefore, neither study provided significant data for intended aims (Carson et al. 2008; Zolfaghari M E et al. 1997). The bibliographic research identified *in vitro* activity of EOs from aromatic plants, including *Cymbopogon citratus, Eucalyptus globulus, Hyssopus officinalis, Mentha piperita, Matricaria recutita, Thymus vulgaris, and Zingiber officinalis*. These, alongside two commercial products and other EOs in their formulations, were considered in this investigation.

This study aimed to identify a new formulation effective against herpetic infections and co‐infections with HSV‐1 and *C. albicans*, which are known for their resistance to treatment (Plotkin et al. 2016a). The antifungal and antiviral effectiveness, along with basophil activation and anti‐inflammatory activity of a new formulation (MIX) were compared to those of the commercial product (LA).

### 4.2 Antifungal Assay

Patients with HSV‐1 are susceptible to *Candida* infections due to shared cellular receptors (Plotkin et al. 2016b). Given the common occurrence of superinfections with *C. albicans* in HSV‐1 patients, one of the aims was to assess the antifungal effectiveness of antiviral EOs selected through a literature review. For this reason, selected EOs were tested against reference *C. albicans* strains, including those resistant to antifungal drugs. EOs active against both HSV‐1 and fungi were *M. piperita, T. vulgaris, E. caryophyllata, and C. martinii* (Table 3). *M. piperita* and *T. vulgaris* show promising fungicidal and virucidal properties based on literature review (Civitelli et al. 2014; Schuhmacher et al. 2003; Schnitzler et al. 2007).

### 4.3 EOs Selection and MIX Formulation

The most important aim of this study was to identify an EOs blend (MIX) with antiviral, antifungal, and anti‐inflammatory properties while ensuring safety. All evaluations of the formulated blend were conducted using a commercial product based on EOs (LA) as the reference control. The commercial product LA showed noteworthy antifungal activity. In fact, in the LA commercial product the EOs are present in the final concentration of 6% v/v, therefore their concentration at the MIC value is equal to 0.24% v/v. However, at the tested concentration, it showed mainly inhibitory rather than fungicidal effects (Table 3). This concentration of OEs is comparable to that of the active concentration of MIX equal to 0.23% v/v. The MIX formulation developed on the basis of the bibliographic results includes *E. caryophyllata* and *C. martini* for their antifungal properties, and *C. camphora* CT 1,8‐cineole and *M. piperita* for their antiviral effects. *E. caryophyllata* and *C. martini* contain eugenol and geraniol, known for their antioxidant and anti‐inflammatory properties (Ulanowska and Olas 2021); Lei et al. 2019), while *C. camphora* CT 1,8‐cineole and *M. piperita*, rich in eucalyptol and menthol respectively are recognized for their antiviral properties (Müller et al. 2016; Zhao et al. 2022).

To ensure the safety of the EOs, adherence to International Fragrance Association (IFRA) standards was ensured. IFRA standards were considered because the European Medicine Agency (EMA) only offers references for individual oils, instead IFRA standards provide for each relevant chemical compound guidelines related to their safe maximal concentration. In fact, IFRA standards establish limits on potentially toxic aromatic substances in cosmetics, forming a globally recognized risk management system. These standards include quantitative restrictions on specific chemical compounds, whether added directly or extracted from natural sources like EO phytocomplexes. Specifically, restrictions for lip products (category 1) were considered. Eugenol in *E. caryophyllata* EO and geraniol in *C. martini* EO are restricted, with eugenol limited to 0.85% and geraniol to 0.45% in the final formulation. All components in the blend adhere to IFRA standards, with both EOs present at 0.4% v/v concentration, ensuring compliance with even lower concentrations than required (Mahendran and Rahman 2020; Neupane et al. 2020; Jain and Sharma 2021).

### 4.4 Antiviral Activity (ELISA Testing)

Experiments on Vero cells confirmed the antiviral effectiveness of EOs in both formulations, in agreement with previous findings (Figure 3). MIX enhances the resilience of target cellsand inhibits viral infection entry, while LA also inhibits intracellular replication. However, this intracellular inhibition was not observed for MIX, likely due to lower tested concentrations compared to LA.

*In vitro* antiviral assays on Vero cells further support the therapeutic potential of these EO‐based formulations. MIX exhibited a protective effect by reinforcing the cellular barrier and preventing HSV‐1 entry, whereas LA not only inhibited viral entry but also reduced intracellular replication—a finding consistent with the known antiviral effectiveness of *E. caryophyllata* (Kiki 2023) and *Cymbopogon martinii* constituents (Okino et al. 2024). Interestingly, the absence of intracellular activity in the MIX formulation may be attributed to the lower concentration of active compounds compared to LA. Previous literature has largely examined individual EOs or their active compounds, but our study provides novel insight into how complex EO blends can achieve synergistic or complementary effects depending on formulation and concentration (Bassolé and Juliani 2012).

### 4.5 Anti‐Inflammatory Potential

In viral and microbial infections, including HSV‐1 infections, an inflammatory response typically occurs to contain and neutralize pathogens. However, excessive inflammation can become harmful. The modulation of this response is crucial for successful therapy. *In vitro* tests on PBMCs from healthy donors studied proinflammatory cytokine expression. Both the LA and MIX formulations, at a middle concentration tested, selectively modulated TNF‐α and IL‐1β expression, key cytokines in HSV‐1‐induced inflammation (Figure 2). This reduction in the proinflammatory response supports previous findings on EOs' ability to modulate cytokine expression (Gandhi et al. 2020). These results suggest the formulations' potential in managing inflammation during HSV‐1 and Candida co‐infections.

In the context of viral and microbial co‐infections such as those involving *HSV‐1* and *Candida albicans*, the inflammatory response plays a dual role: it is essential for host defense but can also cause tissue damage when dysregulated (Inchingolo et al. 2020). Our findings on PBMCs from healthy donors show that both LA and MIX formulations modulate this response in a concentration‐dependent manner, selectively downregulating TNF‐α and IL‐1β expression. This confirms and expands upon previous studies reporting the anti‐inflammatory properties of *Mentha piperita* (Kazemi et al. 2025) and *Cinnamomum camphora* (Xiao et al. 2021).

Notably, our work is among the few to demonstrate this effect in the context of HSV‐1 and *Candida* co‐infections, a clinically relevant but underexplored model (Alicea et al. 2024). By targeting cytokines known to be elevated in HSV‐related inflammation (Kim et al. 2022), these formulations may offer a dual benefit—antimicrobial and immunomodulatory—which has been suggested in past work but not directly demonstrated under co‐infection conditions.

### 4.6 Safety and Allergenicity (BAT Testing)

To evaluate the allergenic activity of formulations, BAT was conducted using fresh blood samples belonging from various donors volunteers (Table 5). The analysis indicates that, at tested concentrations, individual or combined EOs (LA and MIX) are generally safe for topical use. Basophil activation was not induced in most samples, except for one from the allergology department, which reacted to *C. camphora* CT 1,8‐cineole but not to the EOs blend (LA or MIX). These findings stress the importance of adhering to IFRA guidelines for safe formulation and standardized tests to identify potential systemic reactions. This last point is very important because, so far, there are no specifically tests for systemic reactions to EOs but only patch tests for reactions to individual compounds. Additionally, two out of ten tests were inconclusive due to basophil cell death, indicating the need for further standardization, particularly with EOs containing eugenol, a phenolic compound known for cellular toxicity.

To evaluate the allergenic potential of the formulations, BAT was conducted using fresh blood samples from various donors volunteers (Table 5). The analysis indicates that, at the tested concentrations, individual or combined EOs—namely LA and MIX—are generally safe for topical application. Basophil activation was absent in most samples, except for one from the allergology department that responded to *C. camphora* CT 1,8‐cineole, but not to the full EO blends. These findings highlight the relevance of IFRA guidelines for safe EO formulation and emphasize the current gap in standardized systemic allergenicity testing for EOs, which today relies almost exclusively on cutaneous patch testing [REF: IFRA, systemic sensitization reviews].

Our results are in line with previous concerns about the cytotoxicity of phenolic compounds like eugenol (Prashar et al. 2006) as demonstrated by inconclusive BAT results in two samples due to basophil cell death. This underlines the urgent need for standardized protocols that consider both the complexity of EO mixtures and their potential systemic effects—an area rarely addressed in existing literature (Tisserand and Young 2014). This supports the rationale for using EO combinations in clinical formulations, as they can be tailored to target both extracellular and intracellular stages of viral infections. Overall, this study contributes new evidence on the potential of EO‐based formulations in managing co‐infections involving *HSV‐1* and *Candida albicans*, with a focus on safety, immunomodulation, and antiviral activity. By integrating allergenicity assessment, cytokine modulation, and direct antiviral effects, our findings offer a multidimensional perspective rarely addressed in current EO research, and suggest that standardized, well‐characterized EO formulations may represent a promising alternative for topical treatment of mucocutaneous co‐infections.

## 5 Conclusions

The findings endorse the use of EO‐based formulations for treating increasingly common co‐infections of HSV‐1 and *C. albicans*. These formulations not only exhibit antiviral and antifungal properties but also regulate cytokine expression, tempering exaggerated local inflammation. Moreover, standardized BAT diagnostic results suggest that EO blends, when formulated in accordance with international guidelines, are safe for use. Pending further *in vivo* validation of efficacy and safety, these natural formulations could provide valuable support for managing HSV‐1 and *Candida* co‐infections.

## Author Contributions

**Maura Di Vito:** conceptualization, data curation, methodology, validation, writing – original draft, writing – review and editing, **Domiziana Coggiatti:** data curation, formal analysis, investigation, writing – original draft. **Marilena La Sorda:** methodology, data curation, writing – original draft. **Stefania Garzoli:** data curation, formal analysis, methodology, writing – original draft, **Giulia Lombardini:** formal analysis. **Debora Talamonti:** formal analysis. **Scilla Pizzarelli:** methodology, **Abdesselam Zhiri:** writing – review and editing, **Margherita Cacaci:** data curation. **Riccardo Torelli:** methodology. **Maurizio Sanguinetti:** resources, writing – review and editing. **Francesca Bugli:** resources, writing – original draft, writing – review and editing.

## Ethics Statement

The authors have nothing to report.

## Conflicts of Interest

The authors declare no conflicts of interest.

## Data Availability

All data generated or analyzed during this study are included in this published article.
